# Supplementary material for: Mobilome of Apicomplexa Parasites
Source: Genes (Basel). 2022 May 16;13(5):887. doi: 10.3390/genes13050887 (PMC9141347; doi:10.3390/genes13050887)
Supplement: Supplementary file 1 [file genes-13-00887-s001.zip › Table_S1_List_of_Apicomplexa_genomes.pdf]

Table S1. List of Apicomplexa genomes used in this work.

Organism	Strain	Genome assembly (Mb)	Version	Source
Ascogregarina taiwanensis	Taoyuan-1	6.15	GCA_000172235.1	GenBank
Babesia bigemina	Bond	13.84	GCA_000981445.1	GenBank
Babesia bovis	T2Bo	8.18	GCA_000165395.1	GenBank
Babesia divergens	Rouen 1987	9.73	GCA_001077455.2	GenBank
Babesia microti	RI	6.47	GCA_000691945.2	GenBank
Babesia ovata	Miyake	14.45	GCA_002897235.1	GenBank
Besnoitia besnoiti	Bb-Ger1	58.85	GCA_002563875.1	GenBank
Cryptosporidium andersoni	isolate: 30847	9.09	GCA_001865355.1	GenBank
Cryptosporidium baileyi	TAMU-09Q1	8.49	GCA_001593455.1	GenBank
Cryptosporidium bovis	isolate: 42482	9.11	GCA_009768925.1	GenBank
Cryptosporidium cuniculus	UKCU2	9.18	GCA_004337835.1	GenBank
Cryptosporidium hominis	UdeA01	9.07	GCA_002223825.1	GenBank
Cryptosporidium meleagridis	UKMEL1	8.97	GCA_001593445.1	GenBank
Cryptosporidium muris	RN66	9.25	GCA_000006515.1	GenBank
Cryptosporidium parvum	Iowa_II	9.1	GCA_000165345.1	GenBank
Cryptosporidium ryanae	isolate: 45019	9.06	GCA_009792415.1	GenBank
Cryptosporidium tyzzeri	isolate: UGA_55	9.02	GCA_007210665.1	GenBank
Cryptosporidium ubiquitum	isolate: 39726	8.97	GCA_001865345.1	GenBank
Cryptosporidium viatorum	isolate: UKVIA1	9.26	GCA_004337795.1	GenBank
Cyclospora cayetanensis	isolate: NF1_C8	44.36	GCA_002999335.1	GenBank
Cystoisospora suis	Wien I	83.64	GCA_002600585.1	GenBank
Cytauxzoon felis	Winnie	9.11	release-47	PiroplasmaDB
Eimeria acervulina	Houghton	45.83	GCA_000499425.1	GenBank
Eimeria brunetti	Houghton	66.89	GCA_000499725.1	GenBank
Eimeria falciformis	Bayer Haberkorn 1970	43.67	GCA_002271815.1	GenBank
Eimeria maxima	Weybridge	45.98	GCA_000499605.1	GenBank
Eimeria mitis	Houghton	72.24	GCA_000499745.2	GenBank
Eimeria necatrix	Houghton	55.01	GCA_000499385.1	GenBank
Eimeria nieschulzi	isolate: Landers	62.83	GCA_000826945.1	GenBank
Eimeria praecox	Houghton	60.08	GCA_000499445.1	GenBank
Eimeria tenella	Houghton	51.86	GCA_000499545.1	GenBank
Gregarina niphandrodes	-	14.01	GCA_000223845.4	GenBank
Haemoproteus tartakovskyi	SISKIN1	23.21	GCA_001625125.1	GenBank
Hammondia hammondi	HhCatGer041	51.5	GCA_000447165.1	GenBank

Neospora caninum	Liverpool	57.55	GCA_000208865.2	GenBank
Plasmodium adleri	G01	22.19	release-47	PlasmoDB
Plasmodium berghei	ANKA	18.78	GCA_900002375.2	GenBank
Plasmodium billcollinsi	G01	23.11	release-47	PlasmoDB
Plasmodium blacklocki	G01	21.84	release-47	PlasmoDB
Plasmodium brasilianum	Bolivian I	30.09	GCA_001885115.2	GenBank
Plasmodium chabaudi chabaudi	AS	18.97	GCA_900002335.2	GenBank
Plasmodium coatneyi	Hackeri	27.69	GCA_001680005.1	GenBank
Plasmodium cynomolgi	M	30.66	release-47	PlasmoDB
Plasmodium falciparum	3D7	23.33	GCA_000002765.3	GenBank
Plasmodium fragile	Nilgiri	25.91	GCA_000956335.1	GenBank
Plasmodium gaboni	G01	21.38	release-47	PlasmoDB
Plasmodium gallinaceum	8A	25.03	GCA_900005855.1	GenBank
Plasmodium gonderi	ATCC 30045	33.01	GCA_002157705.1	GenBank
Plasmodium inui	San Antonio 1	27.41	GCA_000524495.1	GenBank
Plasmodium knowlesi	Malayan Pk1 (A+)	24.77	GCA_002140095.1	GenBank
Plasmodium malariae	UG01	33.62	GCA_900090045.1	GenBank
Plasmodium ovale	PocGH01	33.48	GCA_900090035.2	GenBank
Plasmodium praefalciparum	G01	24.96	release-47	PlasmoDB
Plasmodium reichenowi	G01	24.47	GCA_900097025.1	GenBank
Plasmodium relictum	SGS1	22.61	GCA_900005765.1	GenBank
Plasmodium vinckei vinckei	-	18.37	GCA_900681995.1	GenBank
Plasmodium vivax	P01	29.04	GCA_900093555.1	GenBank
Plasmodium yoelii	17X	23.08	GCA_900002385.2	GenBank
Sarcocystis neurona	SN3	124.4	GCA_000727475.1	GenBank
Theileria annulata	Ankara	8.36	GCA_000003225.1	GenBank
Theileria equi	WA	11.67	GCA_000342415.1	GenBank
Theileria orientalis	Shintoku	9.01	GCA_000740895.1	GenBank
Theileria parva	Muguga	8.35	GCA_000165365.1	GenBank
Toxoplasma gondii	ME49	65.67	GCA_000006565.2	GenBank
