# Supplementary material for: Mobilome of Apicomplexa Parasites
Source: Genes (Basel). 2022 May 16;13(5):887. doi: 10.3390/genes13050887 (PMC9141347; doi:10.3390/genes13050887)
Supplement: Supplementary file 1 [file genes-13-00887-s001.zip › Table_S2_Classification_of_TE_models.pdf]

Table S2. Classification of TE models, according to TE class, Dfam, SwissProt and InterPro

ID	Length	General classif.	TE class	Dfam			SwissProt			InterPro		
Atai_2	3418	**LTR/Gypsy**	LTR	DF0001544.2	BLASTOPIA_I/Gypsy	1.30E-32	Q8I7P9	Retrovirus-related Pol polyprotein from	3.56E-56	PTHR24559	TRANSPOSON TY3-I GAG-PO	6.40E-74
Atai_3	2691	**LTR/Gypsy**	LTR	DF0001544.3	BLASTOPIA_I/Gypsy	1.90E-43	Q8I7P9	Retrovirus-related Pol polyprotein from	5.97E-67	PTHR24559	TRANSPOSON TY3-I GAG-PO	4.90E-87
Atai_4	1990	**LTR/Gypsy**	unclear	DF0004565.1	Halyomorpha_halys-1_family-27	7.50E-15	P04323	Retrovirus-related Pol polyprotein from	2.20E-11	cd09274	RNase_HI_RT_Ty3	7.98E-27
Atai_6	1480	Unknown	LINE	-	-	-	-	-	-	cd00303	retropepsin_like	1.52E-09
Bdiv_22	735	Unknown	LINE	-	-	-	-	-	-	PTHR35450	REVERSE TRANSCRIPTASE I	2.60E-12
Bova_19	2078	Unknown	LINE	-	-	-	-	-	-	PF00078	Reverse transcriptase (RNA-de	1.90E-26
Ccay_21	1535	Unknown	unclear	-	-	-	-	-	-	cd00303	retropepsin_like	2.03E-18
Ccay_36	1023	**LTR/Gypsy**	unclear	DF0004348.1	Halyomorpha_halys-1_family-23	7.50E-15	Q7LHG5	Transposon Ty3-I Gag-Pol polyprotein	4.76E-18	cd01647	RT_LTR	1.08E-34
Ccay_77	427	**LTR/Gypsy**	LINE	DF0002684.1	Gypsy107-I_Dr	2.40E-16	P04323	Retrovirus-related Pol polyprotein from	7.83E-08	G3DSA:3.30.70.270	Reverse transcriptase/Diguanyl	7.80E-11
Ccay_87	346	Unknown	LINE	-	-	-	-	-	-	PF03732	Retrotransposon gag protein	8.40E-08
Ccay_93	294	**LTR/Gypsy**	DNA	-	-	-	Q99315	Transposon Ty3-G Gag-Pol polyprotein	3.38E-10	PF17921	Integrase zinc binding domain	1.50E-19
Csui_108	553	**LTR/Gypsy**	LTR	DF0002844.1	Gypsy-30-I_DR	6.60E-26	Q7LHG5	Transposon Ty3-I Gag-Pol polyprotein	4.41E-30	G3DSA:3.30.70.270	Reverse transcriptase/Diguanyl	7.80E-48
Csui_11	1904	Unknown	Retro	-	-	-	-	-	-	PF03732	Retrotransposon gag protein	7.30E-12
Csui_125	478	Unknown	LINE	-	-	-	-	-	-	SSF57756	Retrovirus zinc finger-like doma	4.71E-05
Csui_20	1483	Unknown	LTR	-	-	-	-	-	-	cd00303	retropepsin_like	6.50E-07
Csui_3	3775	Unknown	LTR	-	-	-	-	-	-	cd00303	retropepsin_like	4.43E-10
Csui_35	1206	**LTR/Gypsy**	Retro	DF0002541.1	Gypsy72-I_DR	1.00E-23	Q99315	Transposon Ty3-G Gag-Pol polyprotein	1.61E-51	PTHR24559	TRANSPOSON TY3-I GAG-PO	5.90E-89
Csui_42	1099	Unknown	LTR	-	-	-	-	-	-	PTHR34482	DNA DAMAGE-INDUCIBLE PR	2.90E-12
Csui_44	1083	Unknown	LTR	-	-	-	-	-	-	SSF50630	Acid proteases	3.02E-05
Csui_48	1054	Unknown	LTR	-	-	-	-	-	-	cd18978	CD_DDE_transposase_like	5.89E-05
Csui_84	695	Unknown	LTR	-	-	-	-	-	-	cd00303	retropepsin_like	3.81E-16
Csui_85	690	Unknown	Retro	-	-	-	-	-	-	PF17921	Integrase zinc binding domain	5.80E-11
Csui_95	640	**LTR/Gypsy**	Retro	DF0002669.2	Gypsy-173_DR-I	1.30E-07	-	-	-	-	-	-
Eace_10	1749	**LTR/Gypsy**	Retro	DF0003190.1	Gypsy-194_DR-I	1.10E-28	Q7LHG5	Transposon Ty3-I Gag-Pol polyprotein	2.77E-46	PTHR24559	TRANSPOSON TY3-I GAG-PO	1.10E-79
Eace_102	451	Unknown	LTR	-	-	-	-	-	-	G3DSA:4.10.60.10	Acid Proteases	4.40E-12
Eace_38	1057	**LTR/Gypsy**	LTR	DF0002669.2	Gypsy-173_DR-I	1.70E-12	Q99315	Transposon Ty3-G Gag-Pol polyprotein	1.76E-13	PTHR24559	TRANSPOSON TY3-I GAG-PO	5.50E-25
Eace_42	1034	**LTR/Gypsy**	unclear	DF0002513.2	Gypsy-168-I_DR	5.40E-26	P20825	Retrovirus-related Pol polyprotein from	4.44E-40	cd01647	RT_LTR	9.83E-62
Eace_56	814	Unknown	unclear	-	-	-	-	-	-	cd00303	retropepsin_like	1.46E-14
Eace_64	707	**LTR/Gypsy**	LINE	DF0002062.1	RLTR20C2_MM/Gypsy	1.40E-08	-	-	-	-	-	-
Eace_70	633	**LTR/Gypsy**	LINE	DF0003150.2	Gypsy172-I_Dr	6.40E-25	P20825	Retrovirus-related Pol polyprotein from	9.12E-31	cd01647	RT_LTR	7.68E-38
Ebru_16	2009	Unknown	LTR	-	-	-	-	-	-	G3DSA:4.10.60.10	Acid Proteases	1.30E-12
Ebru_31	1362	Unknown	LINE	-	-	-	-	-	-	cd00303	retropepsin_like	6.77E-11
Ebru_34	1269	Unknown	LINE	-	-	-	-	-	-	cd00303	retropepsin_like	6.26E-11
Ebru_57	954	Unknown	LTR	-	-	-	-	-	-	PTHR46148	CHROMO DOMAIN-CONTAINI	1.40E-17
Ebru_7	3254	**LTR/Gypsy**	Retro	DF0001532.2	ACCORD_I/Gypsy	1.20E-23	P20825	Retrovirus-related Pol polyprotein from	1.02E-41	G3DSA:3.30.70.270	Reverse transcriptase/Diguanyl	3.80E-62
Efal_129	570	**LTR/Gypsy**	LINE	DF0003150.2	Gypsy172-I_Dr	1.60E-17	Q7LHG5	Transposon Ty3-I Gag-Pol polyprotein	1.32E-38	PTHR24559	TRANSPOSON TY3-I GAG-PO	3.00E-52
Efal_132	549	**LTR/Gypsy**	LINE	DF0003150.2	Gypsy172-I_Dr	4.50E-16	Q7LHG5	Transposon Ty3-I Gag-Pol polyprotein	4.60E-20	G3DSA:3.30.420.10	Ribonuclease H superfamily	1.00E-37
Efal_145	506	Unknown	LTR	-	-	-	-	-	-	PF17921	Integrase zinc binding domain	3.50E-10
Efal_153	489	**LTR/Gypsy**	DNA	DF0001532.2	ACCORD_I/Gypsy	6.90E-27	Q7LHG5	Transposon Ty3-I Gag-Pol polyprotein	8.99E-39	cd01647	RT_LTR	1.78E-58
Efal_162	461	Unknown	LTR	-	-	-	-	-	-	cd00303	retropepsin_like	3.44E-16
Efal_172	444	**LTR/Gypsy**	DNA	DF0004438.1	Halyomorpha_halys-1_family-19	1.10E-29	Q7LHG5	Transposon Ty3-I Gag-Pol polyprotein	1.20E-37	cd01647	RT_LTR	6.82E-72
Efal_180	428	Unknown	LINE	-	-	-	-	-	-	PF08284	Retroviral aspartyl protease	2.40E-10
Efal_183	420	**LTR/Gypsy**	LINE	DF0004565.1	Halyomorpha_halys-1_family-27	5.60E-08	P20825	Retrovirus-related Pol polyprotein from	7.08E-12	cd09274	RNase_HI_RT_Ty3	2.71E-34
Efal_193	398	PF00385	LINE	-	-	-	Q04832	DNA-binding protein HEXBP	9.85E-07	SSF57756	Retrovirus zinc finger-like doma	1.32E-10
Efal_205	370	**LTR/Gypsy**	Retro	DF0004565.1	Halyomorpha_halys-1_family-27	1.20E-10	P10401	Retrovirus-related Pol polyprotein from	7.43E-21	cd09274	RNase_HI_RT_Ty3	9.16E-49
Efal_209	362	**LTR/Gypsy**	DNA	DF0004973.1	Halyomorpha_halys-1_family-63	4.50E-08	P31792	Pol polyprotein (Fragment)	3.83E-13	G3DSA:3.30.70.270	Reverse transcriptase/Diguanyl	4.10E-18

Efal_210	358	**LTR/Gypsy**	Retro	-	-	-	P0CT43	Transposon Tf2-8 polyprotein	2.11E-09	PF17921	Integrase zinc binding domain	1.10E-16
Efal_242	309	**LTR/Gypsy**	LTR	DF0001608.2	GTWIN_I/Gypsy	1.70E-10	P04323	Retrovirus-related Pol polyprotein from	1.11E-10	PTHR24559	TRANSPOSON TY3-I GAG-PO	3.10E-19
Efal_247	297	**LTR/Gypsy**	LINE	DF0002733.1	Gypsy89-I_DR	4.80E-09	Q7LHG5	Transposon Ty3-I Gag-Pol polyprotein	1.58E-07	PTHR24559	TRANSPOSON TY3-I GAG-PO	6.20E-12
Efal_275	251	**LTR/Gypsy**	LINE	-	-	-	P31792	Pol polyprotein (Fragment)	7.54E-12	PF17919	RNase H-like domain found in r	2.30E-18
Efal_289	232	Unknown	nonLTR	-	-	-	-	-	-	SSF57756	Retrovirus zinc finger-like doma	7.63E-07
Efal_318	178	Unknown	Retro	-	-	-	-	-	-	G3DSA:3.30.70.270	Reverse transcriptase/Diguanyl	1.10E-08
Efal_89	775	**LTR/Gypsy**	LINE	-	-	-	-	-	-	SSF57756	Retrovirus zinc finger-like doma	2.09E-08
Efal_90	758	Unknown	unclear	-	-	-	-	-	-	cd00303	retropepsin_like	1.17E-12
Emax_105	601	**LTR/Gypsy**	LINE	DF0003595.2	Gypsy-210_DR-I	3.30E-11	P0CT43	Transposon Tf2-8 polyprotein	4.27E-32	G3DSA:3.30.70.270	Reverse transcriptase/Diguanyl	8.40E-53
Emax_133	549	Unknown	LTR	-	-	-	-	-	-	G3DSA:2.40.70.10	Acid Proteases 1	4.20E-05
Emax_163	476	Unknown	Retro	-	-	-	-	-	-	cd00303	retropepsin_like	1.71E-12
Emax_299	266	Unknown	Retro	-	-	-	-	-	-	-	-	-
Emax_48	962	**LTR/Gypsy**	Retro	DF0002541.1	Gypsy72-I_DR	1.80E-19	Q99315	Transposon Ty3-G Gag-Pol polyprotein	7.08E-26	G3DSA:3.30.420.10	Ribonuclease H superfamily	1.00E-18
Emax_59	854	**LTR/Gypsy**	DNA	-	-	-	Q7LHG5	Transposon Ty3-I Gag-Pol polyprotein	7.32E-07	G3DSA:3.30.420.10	Ribonuclease H superfamily	9.70E-14
Emit_102	814	**LTR/Gypsy**	DNA	DF0002684.1	Gypsy107-I_Dr	2.70E-29	Q7LHG5	Transposon Ty3-I Gag-Pol polyprotein	9.45E-39	cd01647	RT_LTR	6.83E-78
Emit_148	670	Unknown	LTR	-	-	-	-	-	-	G3DSA:4.10.60.10	Acid Proteases	5.70E-07
Emit_20	1781	**LTR/Gypsy**	LINE	-	-	-	Q99315	Transposon Ty3-G Gag-Pol polyprotein	6.43E-77	PTHR24559	TRANSPOSON TY3-I GAG-PO	3.00E-107
Emit_22	1658	Unknown	LINE	-	-	-	-	-	-	cd00303	retropepsin_like	1.66E-17
Emit_31	1453	**LTR/Gypsy**	LINE	DF0002407.1	Gypsy135-I_DR	1.50E-21	Q99315	Transposon Ty3-G Gag-Pol polyprotein	2.52E-48	PTHR24559	TRANSPOSON TY3-I GAG-PO	2.00E-77
Emit_34	1404	Unknown	LINE	-	-	-	-	-	-	cd18977	CD_POL_like	5.82E-04
Emit_343	394	Unknown	LTR	-	-	-	-	-	-	G3DSA:4.10.60.10	Acid Proteases	9.70E-11
Emit_8	3115	**LTR/Gypsy**	LTR	DF0002669.2	Gypsy-173_DR-I	3.00E-20	P04323	Retrovirus-related Pol polyprotein from	4.76E-83	PTHR24559	TRANSPOSON TY3-I GAG-PO	9.80E-84
Emit_82	898	**LTR/Gypsy**	LTR	DF0002684.1	Gypsy107-I_Dr	2.80E-41	P04323	Retrovirus-related Pol polyprotein from	3.19E-62	cd01647	RT_LTR	5.91E-78
Enie_1	6283	**LTR/Gypsy**	LINE	-	-	-	Q8I7P9	Retrovirus-related Pol polyprotein from	5.27E-48	cd01647	RT_LTR	2.40E-65
Enie_28	1122	**LTR/Gypsy**	LINE	DF0004607.1	Halyomorpha_halys-1_family-23	2.10E-10	-	-	-	G3DSA:3.30.70.270	Reverse transcriptase/Diguanyl	2.80E-05
Enie_3	4181	**LTR/Gypsy**	LTR	DF0002844.1	Gypsy-30-I_DR	8.10E-37	P10394	Retrovirus-related Pol polyprotein from	1.11E-71	PTHR24559	TRANSPOSON TY3-I GAG-PO	6.00E-95
Enie_30	1094	**LTR/Gypsy**	DNA	DF0002940.2	Gypsy145-I_DR	5.40E-23	Q99315	Transposon Ty3-G Gag-Pol polyprotein	1.73E-21	PTHR24559	TRANSPOSON TY3-I GAG-PO	8.00E-45
Enie_5	3671	**LTR/Gypsy**	LTR	DF0001532.2	ACCORD_I/Gypsy	2.20E-59	P04323	Retrovirus-related Pol polyprotein from	6.79E-78	PTHR24559	TRANSPOSON TY3-I GAG-PO	2.50E-78
Enie_9	3178	Unknown	LTR	-	-	-	-	-	-	cd00303	retropepsin_like	5.81E-17
Epra_1	2507	**LTR/Gypsy**	LTR	DF0003150.2	Gypsy172-I_Dr	2.90E-27	P04323	Retrovirus-related Pol polyprotein from	1.89E-78	cd01647	RT_LTR	1.08E-71
Epra_13	963	Unknown	LTR	-	-	-	-	-	-	cd00303	retropepsin_like	3.61E-11
Epra_263	113	Unknown	unclear	-	-	-	-	-	-	G3DSA:2.40.70.10	Acid Proteases 1	6.20E-07
Epra_4	2184	**LTR/Gypsy**	LTR	DF0002669.2	Gypsy-173_DR-I	1.90E-28	Q7LHG5	Transposon Ty3-I Gag-Pol polyprotein	3.83E-51	PTHR24559	TRANSPOSON TY3-I GAG-PO	1.40E-77
Epra_5	2091	Unknown	Retro	-	-	-	-	-	-	G3DSA:3.10.10.10	HIV Type 1 Reverse Transcripta	2.80E-08
Epra_52	544	**LTR/Gypsy**	LTR	-	-	-	Q99315	Transposon Ty3-G Gag-Pol polyprotein	7.28E-14	PF17921	Integrase zinc binding domain	1.20E-16
Epra_56	541	Unknown	LTR	-	-	-	-	-	-	PF13975	gag-polyprotein putative aspart	1.50E-07
Epra_8	1434	**LTR/Gypsy**	LINE	DF0003989.2	Gypsy108-I_Dr	1.80E-19	Q7LHG5	Transposon Ty3-I Gag-Pol polyprotein	1.10E-28	PTHR24559	TRANSPOSON TY3-I GAG-PO	2.50E-53
Epra_87	413	**LTR/Gypsy**	LINE	DF0003384.1	Gypsy-170-I_DR	2.50E-20	P04323	Retrovirus-related Pol polyprotein from	1.20E-14	PTHR24559	TRANSPOSON TY3-I GAG-PO	1.80E-18
Epra_93	400	**LTR/Gypsy**	LINE	DF0004556.1	Halyomorpha_halys-1_family-26	1.90E-16	P04323	Retrovirus-related Pol polyprotein from	3.00E-21	cd09274	RNase_HI_RT_Ty3	1.67E-38
Eten_67	3355	**LTR/Gypsy**	LTR	DF0001608.2	GTWIN_I/Gypsy	1.10E-31	P04323	Retrovirus-related Pol polyprotein from	7.90E-75	cd01647	RT_LTR	1.41E-74
Gnip_100	341	**LTR/Gypsy**	LINE	-	-	-	P20825	Retrovirus-related Pol polyprotein from	8.55E-15	G3DSA:3.30.70.270	Reverse transcriptase/Diguanyl	1.30E-24
Gnip_102	337	**LTR/Gypsy**	LINE	DF0002844.1	Gypsy-30-I_DR	1.20E-15	Q87040	Pro-Pol polyprotein	4.42E-07	G3DSA:3.10.10.10	HIV Type 1 Reverse Transcripta	6.50E-15
Gnip_11	1044	**LTR/Gypsy**	unclear	DF0001544.2	BLASTOPIA_I/Gypsy	1.60E-28	Q7LHG5	Transposon Ty3-I Gag-Pol polyprotein	2.24E-52	cd01647	RT_LTR	8.89E-48
Gnip_119	295	Unknown	LTR	-	-	-	-	-	-	G3DSA:3.30.420.10	Ribonuclease H superfamily	7.70E-11
Gnip_121	292	Unknown	LTR	-	-	-	-	-	-	PF17921	Integrase zinc binding domain	1.80E-14
Gnip_131	270	**LTR/Gypsy**	LINE	DF0001526.2	ZAM_I/Gypsy	7.10E-10	P20825	Retrovirus-related Pol polyprotein from	2.69E-13	G3DSA:3.30.70.270	Reverse transcriptase/Diguanyl	1.10E-23
Gnip_136	264	**LTR/Gypsy**	Retro	-	-	-	-	-	-	PTHR24559	TRANSPOSON TY3-I GAG-PO	2.00E-13
Gnip_138	262	Unknown	Retro	-	-	-	Q9P2P1	Protein NYNRIN	8.96E-06	PF17921	Integrase zinc binding domain	3.30E-16

Gnip_14	1000	**LTR/Gypsy**	Retro	DF0004584.1	Halyomorpha_halys-1_family-18	5.40E-06	P04323	Retrovirus-related Pol polyprotein from	3.10E-28	PTHR24559	TRANSPOSON TY3-I GAG-PO	7.30E-27
Gnip_141	260	**LTR/Gypsy**	LTR	DF0001532.2	ACCORD_I/Gypsy	2.70E-22	clsdivsl	Retrovirus-related Pol polyprotein from	7.15E-15	cd01647	RT_LTR	4.50E-29
Gnip_143	256	Unknown	LTR	-	-	-	-	-	-	G3DSA:3.30.420.10	Ribonuclease H superfamily	8.40E-13
Gnip_144	256	**LTR/Gypsy**	LTR	-	-	-	P31792	Pol polyprotein (Fragment)	1.85E-10	G3DSA:3.30.70.270	Reverse transcriptase/Diguanyl	4.89E-20
Gnip_149	240	Unknown	LTR	-	-	-	-	-	-	G3DSA:2.40.70.10	Acid Proteases	1.30E-07
Gnip_157	224	**LTR/Gypsy**	SINE	-	-	-	-	-	-	PTHR24559	TRANSPOSON TY3-I GAG-PO	1.00E-13
Gnip_16	959	Unknown	LINE	-	-	-	-	-	-	SSF57756	Retrovirus zinc finger-like doma	1.53E-06
Gnip_167	210	**LTR/Gypsy**	LTR	DF0001658.2	Invader6_I/Gypsy	1.20E-11	P03555	Enzymatic polyprotein	6.97E-07	G3DSA:3.30.70.269	Reverse transcriptase/Diguanyl	2.70E-10
Gnip_168	208	**LTR/Gypsy**	LTR	DF0001532.2	ACCORD_I/Gypsy	2.50E-12	P03556	Enzymatic polyprotein	1.82E-13	G3DSA:3.30.70.270	Reverse transcriptase/Diguanyl	2.10E-18
Gnip_17	954	Unknown	Retro	-	-	-	-	-	-	G3DSA:4.10.60.10	Acid Proteases	6.30E-08
Gnip_173	200	Unknown	Retro	-	-	-	-	-	-	PF17921	Integrase zinc binding domain	1.20E-14
Gnip_175	200	Unknown	Retro	-	-	-	-	-	-	G3DSA:3.30.420.10	Ribonuclease H superfamily	8.40E-12
Gnip_18	951	**LTR/Gypsy**	LINE	-	-	-	vegetal	Pro-Pol polyprotein	1.99E-10	G3DSA:3.30.70.270	Reverse transcriptase/Diguanyl	9.00E-30
Gnip_189	179	Unknown	SINE	-	-	-	-	-	-	PF17921	Integrase zinc binding domain	1.50E-10
Gnip_199	164	Unknown	LTR	-	-	-	-	-	-	PF13650	Aspartyl protease	6.20E-07
Gnip_20	876	**LTR/Gypsy**	LTR	DF0002669.2	Gypsy-173_DR-I	1.10E-23	P10394	Retrovirus-related Pol polyprotein from	3.10E-34	PF17919	RNase H-like domain found in r	3.20E-20
Gnip_22	853	**LTR/Gypsy**	LINE	DF0001532.2	ACCORD_I/Gypsy	2.90E-32	P04323	Retrovirus-related Pol polyprotein from	2.32E-43	PTHR24559	TRANSPOSON TY3-I GAG-PO	1.60E-35
Gnip_27	763	**LTR/Gypsy**	Retro	DF0001658.2	Invader6_I/Gypsy	6.00E-16	P10394	Retrovirus-related Pol polyprotein from	3.28E-31	G3DSA:3.30.70.270	Reverse transcriptase/Diguanyl	3.00E-65
Gnip_29	750	Unknown	LINE	-	-	-	-	-	-	G3DSA:3.30.420.10	Ribonuclease H superfamily	9.00E-15
Gnip_30	747	Unknown	LINE	-	-	-	-	-	-	G3DSA:3.30.420.10	Ribonuclease H superfamily	6.90E-16
Gnip_31	734	Unknown	LINE	-	-	-	P03554	Enzymatic polyprotein	1.71E-12	G3DSA:3.30.70.270	Reverse transcriptase/Diguanyl	3.00E-31
Gnip_32	731	**LTR/Gypsy**	Retro	DF0002669.2	Gypsy-173_DR-I	8.50E-16	P10394	Retrovirus-related Pol polyprotein from	8.03E-19	G3DSA:3.30.70.270	Reverse transcriptase/Diguanyl	3.40E-19
Gnip_33	726	**LTR/Gypsy**	LINE	DF0001526.2	ZAM_I/Gypsy	2.50E-13	P04323	Retrovirus-related Pol polyprotein from	1.29E-18	cd01647	RT_LTR	5.31E-43
Gnip_36	688	Unknown	LTR	-	-	-	-	-	-	PF13975	gag-polyprotein putative asparty	1.20E-06
Gnip_45	572	**LTR/Gypsy**	LINE	DF0001544.2	BLASTOPIA_I/Gypsy	1.90E-16	P04323	Retrovirus-related Pol polyprotein from	5.40E-18	cd09274	RNase_HI_RT_Ty3	1.97E-34
Gnip_47	552	**LTR/Gypsy**	LINE	DF0003405.2	Gypsy69-I_DR	1.20E-07	Q4R6I1	Gypsy retrotransposon integrase-like pı	1.39E-07	PF17921	Integrase zinc binding domain	1.10E-17
Gnip_53	523	**LTR/Gypsy**	LINE	DF0002844.1	Gypsy-30-I_DR	4.30E-13	P04323	Retrovirus-related Pol polyprotein from	5.20E-18	cd01647	RT_LTR	2.75E-44
Gnip_59	493	Unknown	LINE	-	-	-	-	-	-	PF17921	Integrase zinc binding domain	8.90E-18
Gnip_6	1579	**LTR/Gypsy**	Retro	DF0001526.2	ZAM_I/Gypsy	6.70E-11	P0CT39	Transposon Tf2-6 polyprotein	7.54E-36	PTHR24559	TRANSPOSON TY3-I GAG-PO	3.30E-74
Gnip_60	486	Unknown	LINE	-	-	-	Q09575	Uncharacterized protein K02A2.6	2.06E-08	G3DSA:3.30.420.10	Ribonuclease H superfamily	2.10E-25
Gnip_61	479	Unknown	LTR	-	-	-	-	-	-	SSF57756	Retrovirus zinc finger-like doma	3.14E-06
Gnip_62	479	**LTR/Gypsy**	LTR	DF0001532.2	ACCORD_I/Gypsy	8.80E-07	P04323	Retrovirus-related Pol polyprotein from	8.94E-27	cd01647	RT_LTR	1.00E-32
Gnip_63	476	Unknown	Retro	-	-	-	-	-	-	G3DSA:3.10.10.10	HIV Type 1 Reverse Transcripta	3.80E-05
Gnip_64	468	**LTR/Gypsy**	LTR	DF0002669.2	Gypsy-173_DR-I	1.80E-07	Q9UR07	Transposon Tf2-11 polyprotein	1.84E-07	G3DSA:3.30.70.270	Reverse transcriptase/Diguanyl	9.70E-19
Gnip_70	436	**LTR/Gypsy**	LINE	DF0001544.2	BLASTOPIA_I/Gypsy	4.70E-07	P04323		1.16E-09	PF17919	RNase H-like domain found in r	2.60E-25
Gnip_73	424	**LTR/Gypsy**	LINE	DF0004631.1	Halyomorpha_halys-1_family-28	2.10E-08	P0CT43	Transposon Tf2-8 polyprotein	9.75E-08	PTHR24559	TRANSPOSON TY3-I GAG-PO	1.70E-13
Gnip_80	401	**LTR/Gypsy**	LINE	DF0001532.2	ACCORD_I/Gypsy	4.40E-07	P04323	Retrovirus-related Pol polyprotein from	6.82E-08	PF17917	RNase H-like domain found in r	5.40E-20
Gnip_83	395	Unknown	LINE	-	-	-	-	-	-	PF13975	gag-polyprotein putative asparty	4.70E-08
Gnip_84	395	**LTR/Gypsy**	unclear	DF0001544.2	BLASTOPIA_I/Gypsy	2.10E-08	P20825	Retrovirus-related Pol polyprotein from	3.26E-09	PF17919	RNase H-like domain found in r	1.00E-18
Gnip_86	380	Unknown	LTR	-	-	-	-	-	-	G3DSA:3.30.420.10	Ribonuclease H superfamily	4.70E-22
Gnip_97	344	**LTR/Gypsy**	Retro	DF0004565.1	Halyomorpha_halys-1_family-27	3.60E-09	P20825	Retrovirus-related Pol polyprotein from	9.04E-16	cd09274	RNase_HI_RT_Ty3	1.56E-33
Pgal_3	6853	**LTR/Gypsy**	DNA	DF0003144.1	Gypsy133-I_DR	1.40E-07	-	-	-	PTHR24559	TRANSPOSON TY3-I GAG-PO	3.10E-40
Pgal_42	788	Unknown	LINE	-	-	-	-	-	-	SSF57756	Retrovirus zinc finger-like doma	5.93E-07
Prel_10	1507	**LTR/Gypsy**	LINE	-	-	-	-	-	-	G3DSA:3.30.420.10	Ribonuclease H superfamily	3.90E-35
Prel_3	2655	**LTR/Gypsy**	LINE	-	-	-	-	-	-	PTHR24559	TRANSPOSON TY3-I GAG-PO	2.00E-72
Prel_4	2280	**LTR/Gypsy**	LINE	DF0003144.1	Gypsy133-I_DR	4.20E-09	-	-	-	G3DSA:3.30.70.270	Reverse transcriptase/Diguanyl	1.70E-30
Prel_8	1802	Unknown	LTR	-	-	-	-	-	-	cd00303	retropepsin_like	2.21E-10
Tequ_3	4426	**LINE**	LTR	-	-	-	Q03274	Retrovirus-related Pol polyprotein from	-	cd01650	RT_nLTR_like	4.18E-39

api_157	7204	**LTR/Gypsy**	LTR	DF0002844.1	Gypsy-30-I_DR	7.20E-37	P10394	Retrovirus-related	6.07E-79	PTHR24559	TRANSPOSON TY3-I GAG-PO	7.40E-122
api_165	6611	**LTR/Gypsy**	LTR	DF0002669.2	Gypsy-173_DR-I	1.00E-18	P04323	Retrovirus-related Pol polyprotein from	1.53E-79	PTHR24559	TRANSPOSON TY3-I GAG-PO	1.20E-87
api_181	4980	**LTR/Gypsy**	LTR	DF0003989.2	Gypsy108-I_Dr	8.20E-28	P04323	Retrovirus-related Pol polyprotein from	4.02E-74	cd01647	RT_LTR	1.90E-69
api_194	4356	**LINE**	LTR	-	-	-	P16423	Retrovirus-related Pol polyprotein from	1.11E-14	cd01650	RT_nLTR_like	7.90E-35
api_207	3684	Unknown	LTR	-	-	-	-	-	-	cd00303	retropepsin_like	1.09E-16
api_213	3430	**LTR/Gypsy**	LTR	DF0002844.1	Gypsy-30-I_DR	6.70E-24	P10394	Retrovirus-related Pol polyprotein from	8.53E-48	PTHR24559	TRANSPOSON TY3-I GAG-PO	1.30E-15
api_231	2659	**LTR/Gypsy**	DNA	DF0002669.2	Gypsy-173_DR-I	3.00E-12	Q7LHG5	Transposon Ty3-I Gag-Pol polyprotein	7.81E-37	PTHR24559	TRANSPOSON TY3-I GAG-PO	3.40E-43
api_254	1885	**LTR/Gypsy**	DNA	DF0003405.2	Gypsy69-I_DR	2.50E-24	P0CT43	Transposon Tf2-8 polyprotein	6.56E-46	PTHR24559	TRANSPOSON TY3-I GAG-PO	2.30E-42
api_318	809	Unknown	DNA	-	-	-	-	-	-	PTHR46148	CHROMO DOMAIN-CONTAINI	1.90E-17
