# Supplementary material for: Mobilome of Apicomplexa Parasites
Source: Genes (Basel). 2022 May 16;13(5):887. doi: 10.3390/genes13050887 (PMC9141347; doi:10.3390/genes13050887)
Supplement: Supplementary file 1 [file genes-13-00887-s001.zip › Table_S3_Classification_of_LTRpred_results.pdf]

Table S3. Classification of LTRpred TEs with SwissProt / InterPro

Contig_coor_LTR	Contig_coor_int	Species	Length	Classification	Best Swissprot / InterPro result		
HG682261.1_360_4695	HG682261.1_618_4430	Emit	4336	LTR/Gypsy	sp|Q99315|YG31B_YEAST	Transposon Ty3-G Gag-Pol poly	1.39E-50
JRZD01020364.1_8198_12708	JRZD01020364.1_8373_12536	Enie	4511	LTR/Gypsy	sp|Q99315|YG31B_YEAST	Transposon Ty3-G Gag-Pol poly	1.86E-56
HG710235.1_73348_77934	HG710235.1_73459_77810	Ebru	4587	LTR/Gypsy	sp|P0CT34|TF21_SCHPO	Transposon Tf2-1 polyprotein OS	1.46E-25
CVMU01000455.1_186_4876	CVMU01000455.1_614_4446	Prel	4691	LTR/Gypsy	sp|Q7LHG5|YI31B_YEAST	Transposon Ty3-I Gag-Pol polyp	2.32E-21
HG682436.1_7487_12180	HG682436.1_7897_11766	Emit	4694	LTR/Gypsy	sp|P0CT34|TF21_SCHPO	Transposon Tf2-1 polyprotein OS	1.42E-13
KK211587.1_118_4817	KK211587.1_327_4595	Gnip	4700	unknown	PF00910	RNA_helicase	5.20E-08
KK211459.1_355622_360423	KK211459.1_355766_360299	Gnip	4802	LTR/Gypsy	sp|P0CT34|TF21_SCHPO	Transposon Tf2-1 polyprotein OS	7.48E-19
HG722498.1_4969_9927	HG722498.1_5279_9643	Enec	4959	LTR/Gypsy	sp|Q7LHG5|YI31B_YEAST	Transposon Ty3-I Gag-Pol polyp	5.49E-11
KK211466.1_6705_11865	KK211466.1_6897_11660	Gnip	5161	LTR/Gypsy	sp|Q7LHG5|YI31B_YEAST	Transposon Ty3-I Gag-Pol polyp	2.20E-19
LN835309.1_1687101_1692447	LN835309.1_1687293_1692258	Prel	5347	LTR/Gypsy	sp|P0CT36|TF23_SCHPO	Transposon Tf2-3 polyprotein OS	5.96E-36
HG675721.1_23707_29155	HG675721.1_23814_29041	Eten	5449	LTR/Gypsy	sp|Q7LHG5|YI31B_YEAST	Transposon Ty3-I Gag-Pol polyp	1.01E-20
KK211464.1_184799_190414	KK211464.1_184959_190255	Gnip	5616	LTR/Gypsy	sp|Q7LHG5|YI31B_YEAST	Transposon Ty3-I Gag-Pol polyp	3.78E-06
CVMV01000039.1_64347_70003	CVMV01000039.1_64805_69539	Pgal	5657	LTR/Gypsy	sp|P0CT39|TF26_SCHPO	Transposon Tf2-6 polyprotein OS	9.79E-15
HG711106.1_122748_128497	HG711106.1_122943_128305	Ebru	5750	LTR/Gypsy	sp|P0CT36|TF23_SCHPO	Transposon Tf2-3 polyprotein OS	8.83E-57
KK211477.1_15421_21182	KK211477.1_15538_21066	Gnip	5762	LTR/Gypsy	sp|Q7LHG5|YI31B_YEAST	Transposon Ty3-I Gag-Pol polyp	2.40E-45
HG681795.1_21412_27201	HG681795.1_21539_27073	Emit	5790	unknown	PF17921	Integrase zinc binding domain	1.20E-11
LSRZ01000044.1_26405_32199	LSRZ01000044.1_26786_31810	Htar	5795	LTR/Gypsy	sp|P0CT34|TF21_SCHPO	Transposon Tf2-1 polyprotein OS	2.25E-26
HG723319.1_1448_7265	HG723319.1_1592_7138	Enec	5818	LTR/Gypsy	sp|P0CT42|TF27_SCHPO	Transposon Tf2-7 polyprotein OS	1.56E-30
KK211489.1_33408_39230	KK211489.1_33526_39108	Gnip	5823	LTR/Gypsy	sp|Q99315|YG31B_YEAST	Transposon Ty3-G Gag-Pol poly	4.23E-19
KK211569.1_225_6122	KK211569.1_381_5976	Gnip	5898	LTR/Gypsy	sp|Q99315|YG31B_YEAST	Transposon Ty3-G Gag-Pol poly	6.64E-91
HG681867.1_5646_11629	HG681867.1_6021_11249	Emit	5984	LTR/Gypsy	sp|Q7LHG5|YI31B_YEAST	Transposon Ty3-I Gag-Pol polyp	8.77E-55
CVMV01000032.1_22487_28491	CVMV01000032.1_22594_28387	Pgal	6005	unknown	G3DSA:3.30.70.270	Reverse_transcriptase/Diguanyla	4.50E-08
HG674134.1_769630_775652	HG674134.1_769953_775339	Eten	6023	LTR/Gypsy	sp|P0CT36|TF23_SCHPO	Transposon Tf2-3 polyprotein OS	3.69E-52
HG681136.1_652_6788	HG681136.1_838_6610	Emit	6137	LTR/Gypsy	sp|Q7LHG5|YI31B_YEAST	Transposon Ty3-I Gag-Pol polyp	6.92E-43
HG674134.1_702355_708498	HG674134.1_702585_708275	Eten	6144	LTR/Gypsy	sp|Q7LHG5|YI31B_YEAST	Transposon Ty3-I Gag-Pol polyp	6.26E-46
KK211463.1_199838_206000	KK211463.1_199998_205854	Gnip	6163	LTR/Gypsy	sp|P0CT42|TF27_SCHPO	Transposon Tf2-7 polyprotein OS	1.77E-06
HG681919.1_587_6761	HG681919.1_851_6509	Emit	6175	LTR/Gypsy	sp|Q7LHG5|YI31B_YEAST	Transposon Ty3-I Gag-Pol polyp	2.10E-44
KK211533.1_4649_10838	KK211533.1_5234_10264	Gnip	6190	LTR/Gypsy	sp|P0CT36|TF23_SCHPO	Transposon Tf2-3 polyprotein OS	3.69E-07
KK211515.1_23511_29734	KK211515.1_23957_29293	Gnip	6224	LTR/Gypsy	sp|Q7LHG5|YI31B_YEAST	Transposon Ty3-I Gag-Pol polyp	2.61E-28
NPHX01000623.1_395913_402243	NPHX01000623.1_396037_402110	Efal	6331	unknown	G3DSA:3.30.70.270	Reverse_transcriptase/Diguanyla	1.50E-09
HG675732.1_71265_77601	HG675732.1_71461_77401	Eten	6337	LTR/Gypsy	sp|P10401|POLY_DROME	Retrovirus-related Pol polyprotei	5.97E-63

HG675688.1_292967_299310	HG675688.1_293313_298985	Eten	6344	LTR/Gypsy	sp|Q7LHG5|YI31B_YEAST	Transposon Ty3-I Gag-Pol polyp	1.00E-67
HG675740.1_91006_97371	HG675740.1_91422_96954	Eten	6366	LTR/Gypsy	sp|Q7LHG5|YI31B_YEAST	Transposon Ty3-I Gag-Pol polyp	7.34E-64
KK211458.1_145402_151828	KK211458.1_146232_150987	Gnip	6427	unknown	G3DSA:3.30.70.270	Reverse_transcriptase/Diguanyla	1.80E-13
HG693258.1_401_6894	HG693258.1_808_6495	Epra	6494	LTR/Gypsy	sp|Q7LHG5|YI31B_YEAST	Transposon Ty3-I Gag-Pol polyp	2.24E-45
HG675710.1_21574_28097	HG675710.1_22001_27691	Eten	6524	LTR/Gypsy	sp|Q99315|YG31B_YEAST	Transposon Ty3-G Gag-Pol poly	3.28E-50
KK211481.1_3812_10436	KK211481.1_4052_10197	Gnip	6625	LTR/Gypsy	sp|P0CT36|TF23_SCHPO	Transposon Tf2-3 polyprotein OS	2.00E-18
HG710647.1_3935_10574	HG710647.1_4046_10439	Ebru	6640	LTR/Gypsy	sp|P0CT36|TF23_SCHPO	Transposon Tf2-3 polyprotein OS	2.40E-67
KK211560.1_10_6707	KK211560.1_434_6282	Gnip	6698	LTR/Gypsy	sp|Q99315|YG31B_YEAST	Transposon Ty3-G Gag-Pol poly	1.44E-08
HG710836.1_102985_109698	HG710836.1_103222_109459	Ebru	6714	LTR/Gypsy	sp|Q99315|YG31B_YEAST	Transposon Ty3-G Gag-Pol poly	2.57E-45
HG695846.1_2484_9213	HG695846.1_2858_8839	Epra	6730	LTR/Gypsy	sp|Q9UR07|TF211_SCHPO	Transposon Tf2-11 polyprotein C	1.01E-38
HG711045.1_1381_8118	HG711045.1_1538_7957	Ebru	6738	LTR/Gypsy	sp|P0CT36|TF23_SCHPO	Transposon Tf2-3 polyprotein OS	8.44E-60
HG674448.1_100958_107713	HG674448.1_101219_107463	Eten	6756	LTR/Gypsy	sp|Q7LHG5|YI31B_YEAST	Transposon Ty3-I Gag-Pol polyp	4.81E-55
HG675753.1_105769_112558	HG675753.1_106038_112293	Eten	6790	LTR/Gypsy	sp|Q7LHG5|YI31B_YEAST	Transposon Ty3-I Gag-Pol polyp	8.71E-42
MIGC01003977.1_1981_8772	MIGC01003977.1_2276_8479	Csui	6792	LTR/Gypsy	sp|Q99315|YG31B_YEAST	Transposon Ty3-G Gag-Pol poly	5.01E-83
HG722320.1_4105_10977	HG722320.1_4352_10724	Enec	6873	LTR/Gypsy	sp|Q99315|YG31B_YEAST	Transposon Ty3-G Gag-Pol poly	1.44E-44
CVMV01000004.1_338491_345403	CVMV01000004.1_338612_345285	Pgal	6913	LTR/Gypsy	sp|Q9UR07|TF211_SCHPO	Transposon Tf2-11 polyprotein C	2.22E-20
HG725993.1_56353_63284	HG725993.1_56961_62679	Enec	6932	LTR/Gypsy	sp|P10401|POLY_DROME	Retrovirus-related Pol polyprotei	1.08E-61
HG713571.1_822_7756	HG713571.1_1230_7344	Ebru	6935	LTR/Gypsy	sp|P0CT42|TF27_SCHPO	Transposon Tf2-7 polyprotein OS	2.72E-56
HG673788.1_70160_77206	HG673788.1_70558_76795	Eten	7047	LTR/Gypsy	sp|P0CT36|TF23_SCHPO	Transposon Tf2-3 polyprotein OS	1.15E-66
HG673821.1_293544_300608	HG673821.1_293950_300201	Eten	7065	LTR/Gypsy	sp|Q99315|YG31B_YEAST	Transposon Ty3-G Gag-Pol poly	1.79E-68
HG713059.1_81286_88393	HG713059.1_81693_87986	Ebru	7108	LTR/Gypsy	sp|Q7LHG5|YI31B_YEAST	Transposon Ty3-I Gag-Pol polyp	6.22E-40
HG692652.1_1924_9040	HG692652.1_2304_8664	Epra	7117	LTR/Gypsy	sp|Q99315|YG31B_YEAST	Transposon Ty3-G Gag-Pol poly	7.59E-50
HG681479.1_813_7938	HG681479.1_1059_7701	Emit	7126	LTR/Gypsy	sp|Q7LHG5|YI31B_YEAST	Transposon Ty3-I Gag-Pol polyp	2.91E-18
HG684190.1_7128_14290	HG684190.1_7429_13993	Emit	7163	LTR/Gypsy	sp|Q7LHG5|YI31B_YEAST	Transposon Ty3-I Gag-Pol polyp	4.52E-66
HG684929.1_3496_10674	HG684929.1_3836_10333	Emit	7179	LTR/Gypsy	sp|Q7LHG5|YI31B_YEAST	Transposon Ty3-I Gag-Pol polyp	4.02E-62
HG683691.1_4451_11685	HG683691.1_4862_11273	Emit	7235	LTR/Gypsy	sp|Q7LHG5|YI31B_YEAST	Transposon Ty3-I Gag-Pol polyp	1.75E-66
MIGC01002064.1_13_7295	MIGC01002064.1_141_7174	Csui	7283	LTR/Gypsy	sp|Q7LHG5|YI31B_YEAST	Transposon Ty3-I Gag-Pol polyp	1.94E-33
HG674448.1_264037_271338	HG674448.1_264480_270891	Eten	7302	LTR/Gypsy	sp|Q7LHG5|YI31B_YEAST	Transposon Ty3-I Gag-Pol polyp	3.48E-37
CVMU01000349.1_132_7529	CVMU01000349.1_563_7114	Prel	7398	LTR/Gypsy	sp|Q9UR07|TF211_SCHPO	Transposon Tf2-11 polyprotein C	6.94E-18
HG675655.1_91584_99077	HG675655.1_91863_98814	Eten	7494	LTR/Gypsy	sp|Q7LHG5|YI31B_YEAST	Transposon Ty3-I Gag-Pol polyp	1.58E-43
HG690086.1_28063_35653	HG690086.1_28314_35434	Epra	7591	LTR/Gypsy	sp|P0CT36|TF23_SCHPO	Transposon Tf2-3 polyprotein OS	1.52E-51
HG712368.1_15660_23275	HG712368.1_16038_22891	Ebru	7616	LTR/Gypsy	sp|Q7LHG5|YI31B_YEAST	Transposon Ty3-I Gag-Pol polyp	4.76E-42
HG696922.1_13941_21575	HG696922.1_14383_21124	Epra	7635	LTR/Gypsy	sp|P0CT36|TF23_SCHPO	Transposon Tf2-3 polyprotein OS	9.53E-57

HG711106.1_82247_89939	HG711106.1_82658_89532	Ebru	7693	LTR/Gypsy	sp|Q7LHG5|YI31B_YEAST	Transposon Ty3-I Gag-Pol polyp	3.41E-30
HG710305.1_8156_15908	HG710305.1_8563_15500	Ebru	7753	LTR/Gypsy	sp|P0CT36|TF23_SCHPO	Transposon Tf2-3 polyprotein OS	1.33E-33
HG725809.1_9674_17493	HG725809.1_9809_17368	Enec	7820	LTR/Gypsy	sp|Q7LHG5|YI31B_YEAST	Transposon Ty3-I Gag-Pol polyp	3.59E-20
KK211478.1_167358_175250	KK211478.1_167812_174823	Gnip	7893	unknown	PF17921	Integrase zinc binding domain	2.40E-10
HG673774.1_169918_177897	HG673774.1_170131_177680	Eten	7980	unknown	SSF57756	Retrovirus_zinc_finger-like_doma	2.54E-05
HG710913.1_5041_13061	HG710913.1_5455_12653	Ebru	8021	LTR/Gypsy	sp|Q7LHG5|YI31B_YEAST	Transposon Ty3-I Gag-Pol polyp	1.70E-57
JRZD01019964.1_15170_23207	JRZD01019964.1_15472_22901	Enie	8038	LTR/Gypsy	sp|Q7LHG5|YI31B_YEAST	Transposon Ty3-I Gag-Pol polyp	8.75E-35
HG719210.1_6353_14396	HG719210.1_6505_14249	Emax	8044	LTR/Gypsy	sp|Q7LHG5|YI31B_YEAST	Transposon Ty3-I Gag-Pol polyp	3.51E-20
HG719159.1_29417_37473	HG719159.1_30248_36647	Emax	8057	LTR/Gypsy	sp|Q99315|YG31B_YEAST	Transposon Ty3-G Gag-Pol poly	6.64E-66
HG687582.1_865_9203	HG687582.1_1285_8781	Emit	8339	LTR/Gypsy	sp|Q7LHG5|YI31B_YEAST	Transposon Ty3-I Gag-Pol polyp	2.54E-42
HG723282.1_170019_178368	HG723282.1_170439_177948	Enec	8350	LTR/Gypsy	sp|P0CT36|TF23_SCHPO	Transposon Tf2-3 polyprotein OS	1.54E-36
HG723041.1_37971_46322	HG723041.1_38181_46112	Enec	8352	LTR/Gypsy	sp|Q7LHG5|YI31B_YEAST	Transposon Ty3-I Gag-Pol polyp	1.87E-46
CVMU01000313.1_968_9323	CVMU01000313.1_1070_9204	Prel	8356	unknown	G3DSA:3.30.420.10	Ribonuclease H superfamily	1.80E-15
KK211530.1_4533_13261	KK211530.1_5035_12765	Gnip	8729	unknown	PF17917	RNase_H-like_domain_found_in	2.30E-14
NPHX01000679.1_12963_22350	NPHX01000679.1_13132_22177	Efal	9388	LTR/Gypsy	sp|Q7LHG5|YI31B_YEAST	Transposon Ty3-I Gag-Pol polyp	3.57E-07
PVNT01000011.1_254069_263521	PVNT01000011.1_255574_262003	Ccay	9453	unknown	G3DSA:3.30.70.270	Reverse_transcriptase/Diguanyla	2.80E-09
HG722610.1_45323_55217	HG722610.1_45508_55019	Enec	9895	LTR/Gypsy	sp|Q7LHG5|YI31B_YEAST	Transposon Ty3-I Gag-Pol polyp	2.00E-47
HG683798.1_11218_21221	HG683798.1_11364_21066	Emit	10004	LTR/Gypsy	sp|Q7LHG5|YI31B_YEAST	Transposon Ty3-I Gag-Pol polyp	2.40E-45
HG691322.1_5605_16076	HG691322.1_5776_15918	Epra	10472	LTR/Gypsy	sp|Q7LHG5|YI31B_YEAST	Transposon Ty3-I Gag-Pol polyp	1.96E-47
JRZD01020054.1_262_12654	JRZD01020054.1_448_12459	Enie	12393	LTR/Gypsy	sp|Q7LHG5|YI31B_YEAST	Transposon Ty3-I Gag-Pol polyp	2.17E-84
MIGC01004125.1_358_12922	MIGC01004125.1_488_12804	Csui	12565	LTR/Gypsy	sp|Q7LHG5|YI31B_YEAST	Transposon Ty3-I Gag-Pol polyp	5.31E-57
CVMU01000304.1_11_12577	CVMU01000304.1_266_12308	Prel	12567	LTR/Gypsy	sp|Q9UR07|TF211_SCHPO	Transposon Tf2-11 polyprotein C	4.11E-07
PVNT01000014.1_264697_277627	PVNT01000014.1_265511_276832	Ccay	12931	LTR/Gypsy	sp|Q99315|YG31B_YEAST	Transposon Ty3-G Gag-Pol poly	6.61E-08
PVNT01000001.1_682830_695808	PVNT01000001.1_683557_695078	Ccay	12979	unknown	sp|P04323|POL3_DROME	Retrovirus-related Pol polyprotei	7.93E-07
CVMU01000039.1_1389_15611	CVMU01000039.1_1527_15469	Prel	14223	LTR/Gypsy	sp|P0CT40|TF29_SCHPO	Transposon Tf2-9 polyprotein OS	6.37E-08
HG694242.1_1573_16310	HG694242.1_1680_16206	Epra	14738	LTR/Gypsy	sp|P10401|POLY_DROME	Retrovirus-related Pol polyprotei	2.73E-08
HG692621.1_2039_16944	HG692621.1_2180_16803	Epra	14906	LTR/Gypsy	sp|P20825|POL2_DROME	Retrovirus-related Pol polyprotei	7.52E-52
HG725887.1_92420_110019	HG725887.1_92677_109763	Enec	17600	LTR/Gypsy	sp|Q8I7P9|POL5_DROME	Retrovirus-related Pol polyprotei	1.55E-10
CVMV01000033.1_1087_20130	CVMV01000033.1_3844_17366	Pgal	19044	unknown	sp|Q6XKE6|POLG_PVCV2	Genome polyprotein OS=Petunia	5.49E-09
HG712979.1_2733_21836	HG712979.1_2888_21687	Ebru	19104	LTR/Gypsy	sp|P04323|POL3_DROME	Retrovirus-related Pol polyprotei	8.39E-54
KK211493.1_40397_59666	KK211493.1_40638_59426	Gnip	19270	LTR/Gypsy	sp|Q7LHG5|YI31B_YEAST	Transposon Ty3-I Gag-Pol polyp	1.86E-18
KK211463.1_94483_114767	KK211463.1_94638_114632	Gnip	20285	unknown	sp|P04323|POL3_DROME	Retrovirus-related Pol polyprotei	4.49E-12
