# Supplementary material for: Mobilome of Apicomplexa Parasites
Source: Genes (Basel). 2022 May 16;13(5):887. doi: 10.3390/genes13050887 (PMC9141347; doi:10.3390/genes13050887)
Supplement: Supplementary file 1 [file genes-13-00887-s001.zip › Table_S4_Number_of_TE_alignments_in_Apicomplexa_genomes.pdf]

Table S4. Number of TE alignments in the Apicomplexa genomes.

Names in columns are the first four letters of each taxa, e.g. *Atai is A. taiwanensis*
All Cryptosporidium genomes are represented by column Cspp since we had no results.

Name	Atai	Bbig	Bbov	Bdiv	Bmic	Bova	Bbes	Cspp	Ccay	Csui	Cfel	Eace	Ebru	Efal	Emax	Emit	Enec	Enie	Epra	Eten	Gnip	Htar
api_157										1				8				270				
Pgal_3																						1
api_165									4	1		11	67	2	11	15	564	5	25	365		
Enie_1														5				788				
api_181									21	8		15	141	2	54	40	84	9	50	59		
Tequ_3																						
api_194		67		22		28																
Enie_3														2			1	55		1		
Csui_3										49												
api_207														16				65				
Enie_5									18	2			40	73		2	1	190				
api_213	1									170											2	
Atai_2	30																					
Eten_67									2	1		2	15		1	3	55		1	23		
Ebru_7									7	10		3	85		10	36	46		35	34		
Enie_9														12				114				
Emit_8									28	37			20	7	2	112	7	15	11	3		
Atai_3	23																				5	
api_231										120												
Prel_3																						6
Epra_1									12	14		9	11	1	7	24	14		27	8		
Prel_4																						3
Epra_4									6			7	29	2	12	7	42	1	33	33		
Epra_5									3			13	41		8	19	8		36	2		
Bova_19		9		9		70																
Ebru_16									5			1	74	3		14			6			
Atai_4	18																					
Csui_11										96												
api_254										171												
Prel_8																						1

Name	Hham	Ncan	Padl	Pber	Pbil	Pbla	Pbra	Pcha	Pcoa	Pcyn	Pfal	Pfra	Pgab	Pgal	Pgon	Pinu	Pkno	Pmal	Pova	Ppra	Prei	Prel	Pvin	Pviv	Pyoe	Sneu	Tann	Tequ	Tori	Tpar	Tgon
api_157																															
Pgal_3														1449								90									
api_165																															
Enie_1																															
api_181																															
Tequ_3																												64			
api_194																												1			
Enie_3																															
Csui_3																															
api_207																															
Enie_5																															
api_213																															
Atai_2																															
Eten_67																															
Ebru_7																															
Enie_9																															
Emit_8																															
Atai_3																															
api_231																															
Prel_3														127								172									
Epra_1																															
Prel_4														50								101									
Epra_4																															
Epra_5																															
Bova_19																															
Ebru_16																															
Atai_4																															
Csui_11																															
api_254																															
Prel_8														88								190									

Name	Atai	Bbig	Bbov	Bdiv	Bmic	Bova	Bbes	Cspp	Ccay	Csui	Cfel	Eace	Ebru	Efal	Emax	Emit	Enec	Enie	Epra	Eten	Gnip
Emit_20									783			240	314	8	158	328		30	458		
Eace_10												13	23	2	2	3	5	8	14	3	
Emit_22									13			441	577	7	199	605		11	661		
Gnip_6	2																				18
Ccay_21									738			39	28	6	7	46		10	61		
Prel_10																					
Csui_20										154											
Atai_6	10																				
Emit_31									92			75	99	10	41	142		12	128		
Epra_8										2		2	26		5	12	27	1	41	16	
Emit_34									1			26	15	8	4	196		4	22		
Ebru_31													32						1		
Ebru_34									9			2	40	3	1	4		1	1		
Csui_35										473											
Enie_28									27			15	12	23	4	21		40	43		
Csui_42										100											
Enie_30									6	3		1	16	32		2		134			
Csui_44										78											
Eace_38												3	6	2			4		1	3	
Csui_48										38											
Gnip_11																					19
Eace_42									2	13		7	6	1	2	10	19		2	21	1
Ccay_36									725			55	59	6	10	78		10	93		
Gnip_14	3									6											65
Epra_13												3	13		36	10	3		32	3	
Emax_48									6			1	3	1	10	2	7	3	1	3	
Gnip_16																					35
Ebru_57												72	206		75	104	58		140	38	
Gnip_17																					43
Gnip_18																					76
Emit_82	1								437	10		162	207	7	77	195		15	317		
Gnip_20										7											99
Emax_59									6			62	61		90	52			111		

Name	Htar	Hham	Ncan	Padl	Pber	Pbil	Pbla	Pbra	Pcha	Pcoa	Pcyn	Pfal	Pfra	Pgab	Pgal	Pgon	Pinu	Pkno	Pmal	Pova	Ppra	Prei	Prel	Pvin	Pviv	Pyoe	Sneu	Tann	Tequ	Tori	Tpar	Tgon
Emit_20																																
Eace_10																																
Emit_22																																
Gnip_6																																
Ccay_21																																
Prel_10	1														55								1388									
Csui_20																																
Atai_6																																
Emit_31																																
Epra_8																																
Emit_34																																
Ebru_31																																
Ebru_34																																
Csui_35																																
Enie_28																																
Csui_42																																
Enie_30																																
Csui_44																																
Eace_38																																
Csui_48																																
Gnip_11																																
Eace_42																																
Ccay_36																																
Gnip_14																																
Epra_13																																
Emax_48																																
Gnip_16																																
Ebru_57																																
Gnip_17																																
Gnip_18																																
Emit_82																																
Gnip_20																																
Emax_59																																

Name	Atai	Bbig	Bbov	Bdiv	Bmic	Bova	Bbes	Cspp	Ccay	Csui	Cfel	Eace	Ebru	Efal	Emax	Emit	Enec	Enie	Epra	Eten	Gnip
Gnip_22	10									101		1									57
Eace_56									2			13	6		5	3	7		5	11	
Emit_102									360	4		230	280	8	106	298		13	453		
api_318									1				3	68				1			
Pgal_42																					
Efal_89														17				2			
Gnip_27	6									17											131
Efal_90														17				6			
Gnip_29																					64
Gnip_30																					44
Bdiv_22				66																	
Gnip_31	4									1											43
Gnip_32																					75
Gnip_33																					26
Eace_64										2		20	15		15	8	12		19	1	
Csui_84										68											
Csui_85										81											
Gnip_36																					38
Emit_148																54					
Csui_95										215											
Eace_70									1	10		3	1			2	3	1	2	2	
Emax_105									7	19		1	3	3	6	4	3	3		4	
Gnip_45	1									1											23
Efal_129									5				1	27				4	1		
Csui_108	4									102											13
Gnip_47																					19
Efal_132									3				1	23				8			
Emax_133												1	1		30				5		
Epra_52									64			54	54	3	30	43		7	110		
Epra_56													4			9	4		8	8	
Gnip_53										3											15
Ebru_176												1	40			2	8		6	7	
Efal_145													7	18							

Name	Htar	Hham	Ncan	Padl	Pber	Pbil	Pbla	Pbra	Pcha	Pcoa	Pcyn	Pfal	Pfra	Pgab	Pgal	Pgon	Pinu	Pkno	Pmal	Pova	Ppra	Prei	Prel	Pvin	Pviv	Pyoe	Sneu	Tann	Tequ	Tori	Tpar	Tgon
Gnip_22																																
Eace_56																																
Emit_102																																
api_318																																
Pgal_42	1														59								15									
Efal_89																																
Gnip_27																																
Efal_90																																
Gnip_29																																
Gnip_30																																
Bdiv_22																																
Gnip_31																																
Gnip_32																																
Gnip_33																																
Eace_64																																
Csui_84																																
Csui_85																																
Gnip_36																																
Emit_148																																
Csui_95																																
Eace_70																																
Emax_105																																
Gnip_45																																
Efal_129																																
Csui_108																																
Gnip_47																																
Efal_132																																
Emax_133																																
Epra_52																																
Epra_56																																
Gnip_53																																
Ebru_176																																
Efal_145																																

Name	Atai	Bbig	Bbov	Bdiv	Bmic	Bova	Bbes	Cspp	Ccay	Csui	Cfel	Eace	Ebru	Efal	Emax	Emit	Enec	Enie	Epra	Eten	Gnip	Htar	Hham	Ncan	Padl	Pber	Pbil	Pbla	Pbra	Pcha	Pcoa	Pcyn	Pfal	Pfra	Pgab	Pgal	Pgon	Pinu	Pkno	Pmal	Pova	Ppra	Prei	Prel	Pvin	Pviv	Pyoe	Sneu	Tann	Tequ	Tori	Tpar	Tgon
Gnip_59																					40																																
Efal_153									7	13			2	18				1			1																																
Gnip_60																					51																																
Gnip_61																					14																																
Gnip_62																					34																																
Csui_125										43																																											
Emax_163													1		8	4			1																																		
Gnip_63																					28																																
Gnip_64																					29																																
Efal_162														21																																							
Eace_102												9	23		16	17	8		12	1																																	
Efal_172									11	29		1	8	26			1	14		1																																	
Gnip_70	1																				19																																
Efal_180														25				1																																			
Ccay_77									683			21	29	2	5	46		6	34																																		
Gnip_73																					16																																
Efal_183									1	33		1	3	9		2		10																																			
Epra_87	1								67	1		55	77		31	40		2	98																																		
Gnip_80	3																				16																																
Epra_93									229			141	164	4	65	149		9	237																																		
Efal_193														25				2																																			
Gnip_83																					17																																
Gnip_84																					15																																
Emit_343												1	1			42			2																																		
Gnip_86																					14																																
Efal_205									3	9			5	19				13																																			
Efal_209									2					20			1	14																																			
Efal_210														16				12																																			
Ccay_87									791			2		2				10																																			
Gnip_97	2									1											15																																
Gnip_100																					21																																
Gnip_102																					8																																
Efal_242													1	17		1		2																																			

Name	Atai	Bbig	Bbov	Bdiv	Bmic	Bova	Bbes	Cspp	Ccay	Csui	Cfel	Eace	Ebru	Efal	Emax	Emit	Enec	Enie	Epra	Eten	Gnip	Htar	Hham	Ncan	Padl	Pber	Pbil	Pbla	Pbra	Pcha	Pcoa	Pcyn	Pfal	Pfra	Pgab	Pgal	Pgon	Pinu	Pkno	Pmal	Pova	Ppra	Prei	Prel	Pvin	Pviv	Pyoe	Sneu	Tann	Tequ	Tori	Tpar	Tgon
Efal_247									1					5				4																																			
Gnip_117																					22																																
Gnip_119																					16																																
Ccay_93									539																																												
Gnip_121																					17																																
Gnip_131																					16																																
Emax_299															7	1																																					
Gnip_136																					12																																
Gnip_138																					12																																
Gnip_141	2									13											15																																
Csui_185										47																																											
Gnip_143																					18																																
Gnip_144																					9																																
Efal_275										4				11				1																																			
Csui_190										36																																											
Gnip_149																					26																																
Efal_289														2																																							
Gnip_157																					5																																
Csui_204										5																																											
Efal_296														3																																							
Gnip_167																					7																																
Gnip_168	1									10											9																																
Gnip_173																					18																																
Gnip_174																					12																																
Gnip_175																					15																																
Gnip_189																					13																																
Efal_318														8				1																																			
Gnip_199																					16																																
Gnip_243																					9																																
Epra_263									1			6	6		7	7			11																																		
